# Multifunctional SPIO/DOX-loaded A54 Homing Peptide Functionalized Dextran-g-PLGA Micelles for Tumor Therapy and MR Imaging

**DOI:** 10.1038/srep35910

**Published:** 2016-10-24

**Authors:** Jun-Qing Situ, Xiao-Juan Wang, Xiu-Liang Zhu, Xiao-Ling Xu, Xu-Qi Kang, Jing-Bo Hu, Chen-Ying Lu, Xiao-Ying Ying, Ri-Sheng Yu, Jian You, Yong-Zhong Du

**Affiliations:** 1 College of Pharmaceutical Sciences, Zhejiang University, 866 Yuhangtang Road, Hangzhou 310058, China; 2 Department of Radiology, Second Affiliated Hospital, School of Medicine, Zhejiang University, Hangzhou 310009, China

## Abstract

Specific delivery of chemotherapy drugs and magnetic resonance imaging (MRI) contrast agent into tumor cells is one of the issues to highly efficient tumor targeting therapy and magnetic resonance imaging. Here, A54 peptide-functionalized poly(lactic-co-glycolic acid)-grafted dextran (A54-Dex-PLGA) was synthesized. The synthesized A54-Dex-PLGA could self-assemble to form micelles with a low critical micelle concentration of 22.51 μg. mL^−1^ and diameter of about 50 nm. The synthetic A54-Dex-PLGA micelles can encapsulate doxorubicin (DOX) as a model anti-tumor drug and superparamagnetic iron oxide (SPIO) as a contrast agent for MRI. The drug-encapsulation efficiency was about 80% and the *in vitro* DOX release was prolonged to 72 hours. The DOX/SPIO-loaded micelles could specifically target BEL-7402 cell line. *In vitro* MRI results also proved the specific binding ability of A54-Dex-PLGA/DOX/SPIO micelles to hepatoma cell BEL-7402. The *in vivo* MR imaging experiments using a BEL-7402 orthotopic implantation model further validated the targeting effect of DOX/SPIO-loaded micelles. *In vitro* and *in vivo* anti-tumor activities results showed that A54-Dex-PLGA/DOX/SPIO micelles revealed better therapeutic effects compared with Dex-PLGA/DOX/SPIO micelles and reduced toxicity compared with commercial adriamycin injection.

Hepatocellular carcinoma is an aggressive tumor and the sixth most deadly form of cancer worldwide^1^. Chemotherapy is commonly used to treat cancer patients. However, traditional chemotherapeutic agents exhibit poor specificity to reach tumor tissues and thus can cause serious side effects. Anthracycline anti-tumor drug doxorubicin, which is widely utilized in the clinical therapy, has been approved for treatment in a variety of solid and hematologic tumors^2^,^3^. The exact mechanism of action is thought to interact with DNA by intercalation, including the inhibition of DNA helicases, topoisomerase II and RNA polymerase^4^. The current treatment of doxorubicin is limited to clinical long-term therapy due to the severe side effects, notably the dose-limited cardiotoxicity and myelosuppression^5^. Targeting agents try to change the body distribution pattern of drug, delivery exogenous drug to molecular targets, in order to maximize the efficacy of drug action and reduce the side effects of drugs.

In recent years, polymeric micelles with special core-shell structure have received significant attention for the delivery of hydrophobic antitumor drugs^6^,^7^,^8^, because the inner hydrophobic core can accommodate hydrophobic drugs, while the hydrophilic shell enables the stabilization in an aqueous environment, and they could self-assemble to from amphiphilic block or graft copolymers^9^,^10^. Polymeric micelles, with a size distribution less than 100 nm, can accumulate in tumor tissue via the passive “enhanced permeability and retention (EPR) effect”. On the other hand, polymer micelle can be modified with ligand or antibody to achieve active targeting of tumor tissue^11^,^12^,^13^. Currently, several micellar formulations for anticancer therapy are under clinical evaluation, and a few of them have been FDA approved for use in patients^14^.

With the surface modification, drug delivery system can achieve the purpose of specifically target to particular structures of cell surface. Homing peptides, as modification substance for targeting agents, are widely used in tumor treatment these years^15^,^16^. Homing peptides, with advantages of being easily synthesized, relatively small molecular weights, reversely low cytotoxicity and immunogenicity, degrading *in vivo* to naturally-occurring compounds, may be useful for tumor targeting. Several investigators have successfully discovered cell surface binding peptides using phage-display library methods^17^,^18^. AGKGTPSLETTP peptide (A54), a hepatocarcinoma-binding peptide, has been screened from a phage-display random peptide library, which is the most effective peptide specific to the human hepatoma carcinoma cell line BEL-7402^19^.

Magnetic resonance imaging (MRI), with the advantages of non-invasive, multiparametric imaging and deep soft tissue penetration^20^, has become a powerful technique in cancer diagnosis since it has been approved for clinical use by the FDA in 1985^21^. Tumor-specific targeting MR imaging has large prospect^22^,^23^,^24^ and nanoparticle encapsulating contrast agents can enhance the contrast between normal tissues and tumors^25^. Superparamagnetic iron oxide nanoparticles (SPIONs), because of non-toxicity, biocompatibility and suitable magnetic properties, have been intensively investigated as promising MRI probes^26^,^27^,^28^,^29^. However, the common challenges of SPIONs were insufficient uptake of superparamagnetic iron oxide (SPIO) by specific cells due to instability and low efficiency of internalization^30^. To overcome these problems, polymeric micelles, as the powerful multifunctional platform for drug delivery and the application of diagnosis imaging, were utilized as a SPIO carrier system^31^,^32^,^33^. The strategy incorporating imaging probes and drugs together into polymeric micelles can achieve combining diagnostic, monitoring and therapeutic components into one system. As a result, precise treatment to cancer will be achieved.

In this study, AGKGTPSLETTP peptide (A54), was used as a homing peptide to synthesize A54 peptide functional Dex-PLGA (A54-Dex-PLGA) for specially targeting the human hepatoma cell line BEL-7402^34^,^35^. Using DOX as a model drug, SPIO as a MRI contrast agent, a multifunctional graft micelle delivery system A54-Dex-PLGA/DOX/SPIO was constructed for tumor diagnosis, detection and therapy. The structure, CMC, micelle size and its morphology of A54-Dex-PLGA micelles were investigated. Drug-loading ability, drug encapsulation and *in vitro* release profiles were then evaluated. Cellular uptake, *in vitro* and *in vivo* anti-tumor activities have been further studied. Moreover, *in vitro* MRI and orthotopic implantation model *in vivo* MRI experiments were validated.

## Results and Discussion

### Synthesis and characteristics of A54-Dex-PLGA

**Figure 1:**
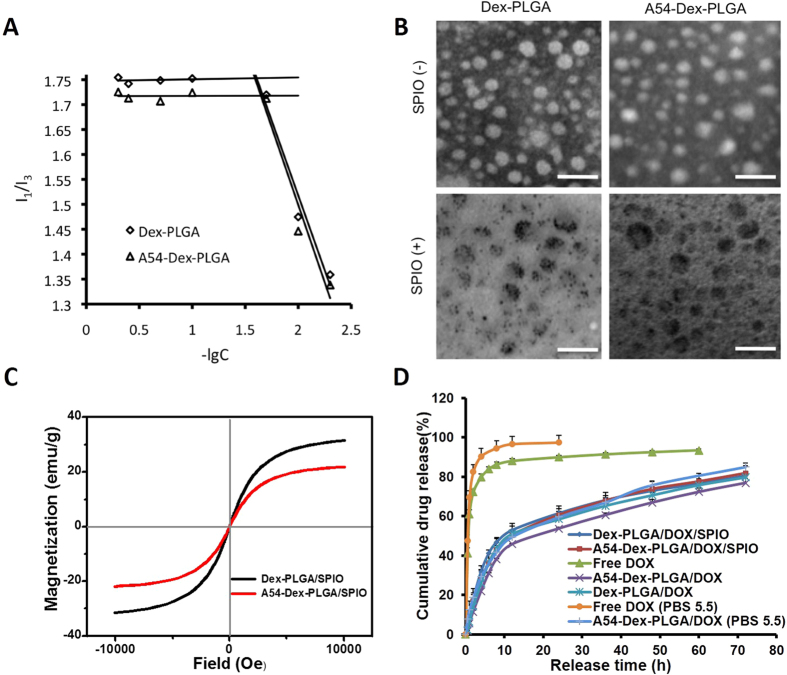
(**A**) Variation of fluorescence intensity ratio for I_1_/I_3_ against logarithm of Dex-PLGA and A54-Dex-PLGA micelles. The unit of concentration was μg mL^−1^. **(B)** Transmission electron microscopy photographs of Dex-PLGA micelles, A54-Dex-PLGA micelles, Dex-PLGA/SPIO micelles and A54-Dex-PLGA/SPIO micelles; scale bar = 0.1 μm. **(C)** Magnetic hysteresis loops of Dex-PLGA/SPIO and A54-Dex-PLGA/SPIO micelles. **(D)** *In vitro* drug release profile of free DOX; Dex-PLGA/DOX and A54-Dex-PLGA/DOX micelles; Dex-PLGA/DOX/SPIO and A54-Dex-PLGA/DOX/SPIO micelles.

A54-Dex-PLGA was successfully synthesized via two-step esterification reaction and chemical structure of A54-Dex-PLGA was confirmed by ^1^H-NMR spectrum^36^. The synthesized Dex-PLGA and A54-Dex-PLGA could self-aggregate to form polymeric micelles in aqueous system with low critical micelles concentration (CMC). Figure 1A showed that the CMC values of Dex-PLGA and A54-Dex-PLGA were 24.70 μg mL^−1^ and 22.51 μg mL^−1^, respectively and the A54 modification had no distinct influence on the formation of micelles.

### Preparation and physicochemical characteristics of DOX/SPIO-loaded Dex-PLGA and A54-Dex-PLGA micelles

SPIO was purchased from Sigma-Aldrich Chemical (USA) and emulsion-solvent evaporation method was utilized to prepared SPIO-loaded Dex-PLGA and A54-Dex-PLGA micelles. A ZETASIZER and a TEM were then employed to evaluate the micelle size and morphology. Particle diameter of Dex-PLGA, A54-Dex-PLGA micelles and SPIO-loaded micelles were listed in Table 1. The results showed that after A54 modification the micelle size increased, which might result from the hydrophilic A54 peptide on the surface of A54-Dex-PLGA micelles toward the water phase. When SPIO was entrapped into the hydrophobic core, the micelle size was smaller than the blank ones, and the size distribution was more uniform (PI < 0.5). It may be due to the hydrophobic interaction between hydrophobic core and SPIO became stronger after SPIO loading which made the micelles more stable. TEM photographs of Dex-PLGA, A54-Dex-PLGA micelles and SPIO-loaded micelles were shown in Fig. 1B. The photographs demonstrated the successful entrapment of SPIO into Dex-PLGA and A54-Dex-PLGA micelles and the particle size of SPIO-loaded micelles was approximately 50 nm. The result was smaller than that from ZETASIZER, which was because the hydrated particle size of micelles would be relatively bigger.

To determine the magnetic property of SPIO-loaded polymeric micelles, the micelles were lyophilized and magnetic properties of the powders were evaluated by vibrating sample magnetometer (VSM) at room temperature. Figure 1C presented typical plots of magnetization versus applied magnetic field and saturation magnetization (Ms) values of Dex-PLGA/SPIO and A54-Dex-PLGA/SPIO polymeric micelles were 31 emu/g Fe and 21 emu/g Fe, respectively, indicating that both SPIO-loaded micelles exhibited good superparamagnetic properties.

DOX/SPIO-loaded Dex-PLGA and A54-Dex-PLGA micelles were further prepared by emulsion-solvent evaporation method. The hydrodynamic diameters, entrapment efficiency (EE%) and drug loading (DL%) of DOX-loaded, DOX/SPIO-loaded micelles were shown in Table 2. When the drug feeding amount was 5%, the Drug Loading of DOX-loaded, DOX/SPIO-loaded micelles exceed 3.5%. Considering the drug loss in preparation process, the Drug Encapsulation Efficiencies of them were found to be approached 80%. And there were no obvious influences for drug loading ability after A54 peptide modification and SPIO encapsulation.

### *In vitro* DOX release from DOX-loaded, DOX and SPIO-loaded micelles

*In vitro* DOX release from DOX-loaded, DOX/SPIO-loaded micelles was carried out by dialysis method using pH 7.2 PBS as dissolution medium. As show in Fig. 1D, the release profiles of DOX-loaded, DOX/SPIO-loaded micelles were mostly similar, which implied that the encapsulation of SPIO and the modification of A54 peptide had almost no effects on the drug release behaviors. The curves revealed a typical two-phase pattern consisting of a premier fast release in 12 h followed by sustained release for a prolonged time. There are approximately 80% drugs released after 72 h. Furthermore, to reflect intracellular acidic environment of the tumor, the drug release behavior of free DOX and A54-Dex-PLGA/DOX in pH 5.5 PBS were performed. The results demonstrated that both free DOX and A54-Dex-PLGA/DOX exhibited faster drug release in pH 5.5 PBS compared with pH 7.2 PBS. This was probably attributed to the structure of DOX, which exhibited higher saturation solubility in pH 5.5 PBS due to the existence of amino group.

### Cellular internalization ability of the micelles

**Figure 2:**
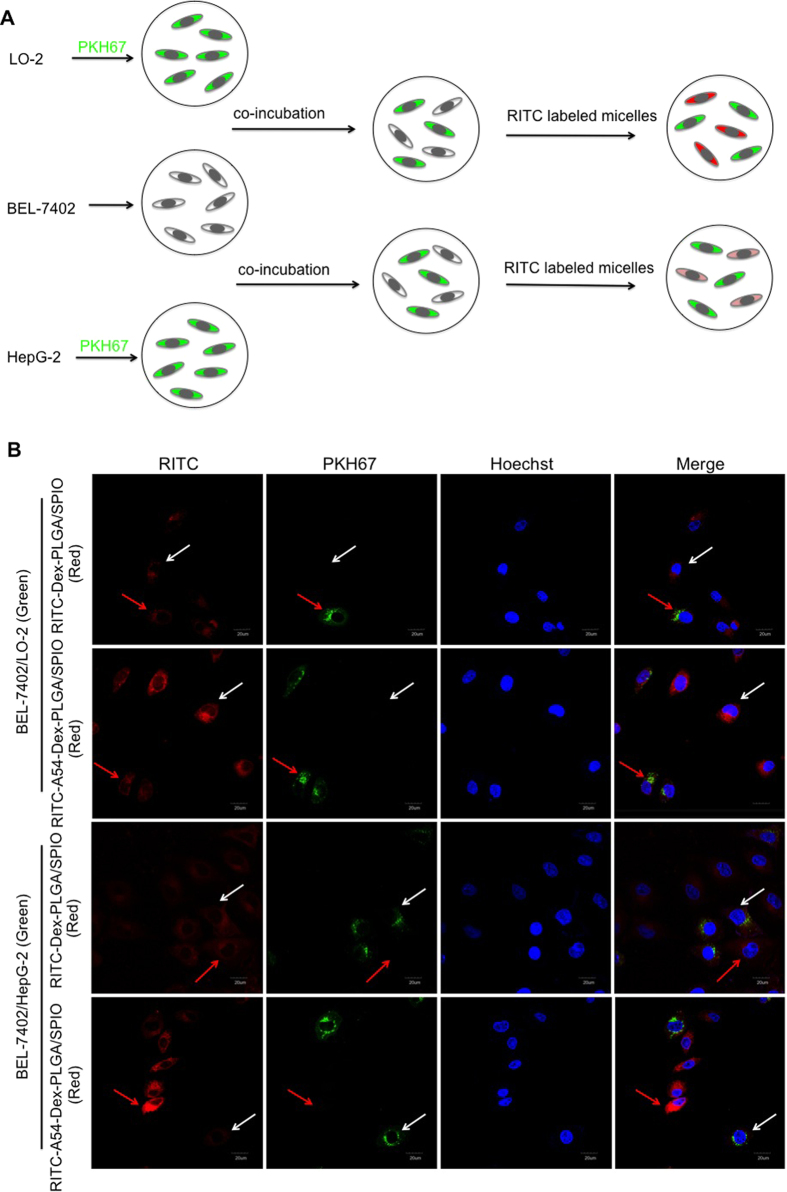
(**A**) Schematic diagram of cellular competitive uptake of Dex-PLGA and A54-Dex-PLGA micelles. **(B)** Confocal microscopy images of RITC labeled micelles for 1 h. HepG2 and LO2 cells (the cytoplasmic membrane labeled with PKH67 fluorescent linker, Green) co-cultured with BEL-7402 cells were incubated with RITC-Dex-PLGA/SPIO and RITC-A54-Dex-PLGA/SPIO micelles (Red). The nucleus were all stained with Hoechst 33342.

In order to directly investigate the targeting ability of A54-Dex-PLGA micelles toward BEL-7402 cell line, cellular competitive uptake of RITC labeled SPIO-loaded micelles on HepG2/BEL-7402 and LO2/BEL-7402 cells co-cultured systems (Fig. 2A) were observed by a confocal microscopy. More efficient uptake of A54-Dex-PLGA/SPIO micelles on BEL-7402 cells compared with HepG2 and LO2 cells could be observed in Line 2 and line 4 of Fig. 2B, which indicated that A54-Dex-PLGA/SPIO micelles could specifically target BEL-7402 cells. While Dex-PLGA/SPIO micelles showed weaker uptake either on the HepG2/BEL-7402 or LO2/BEL-7402 co-cultured systems and no obvious difference was observed (Line 1 and line 3 in Fig. 2B). The cellular competitive uptake data confirmed specific binding activity of A54-Dex-PLGA/SPIO micelles to BEL-7402 cells, due to the presence of an abundant cell surface marker that may have high expression on BEL-7402 cells then taken up by cells via receptor-mediated endocytosis^19^. These results established that the A54-Dex-PLGA/SPIO micelles retain their A54 peptide combining capacity and specificity to BEL-7402 cells, and may be utilized as a prospective active-targeting carrier to deliver drugs or contrast agents^37^,^38^,^39^.

**Figure 3:**
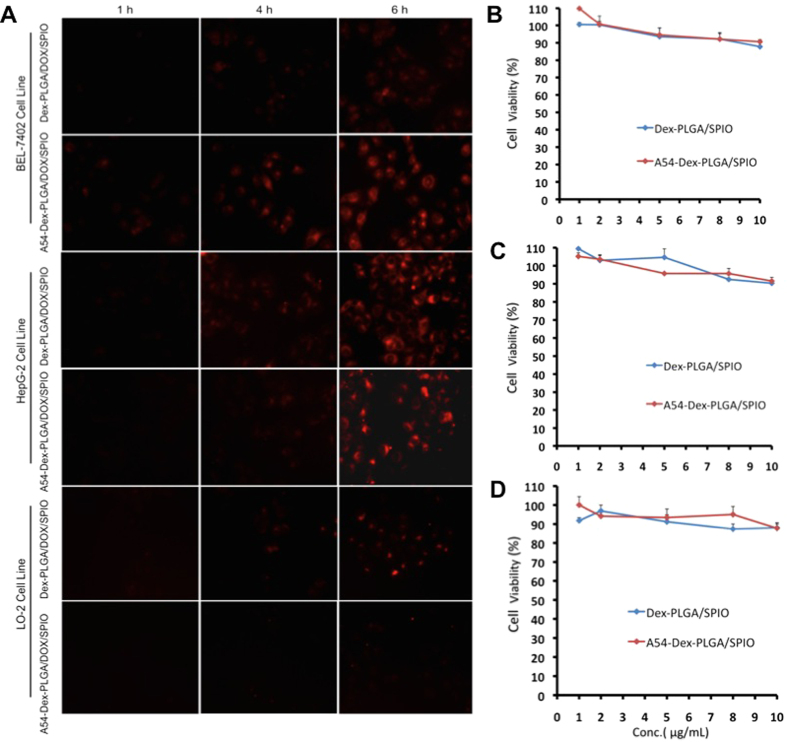
(**A**) *In vitro* cellular uptake of DOX/SPIO-loaded micelles in different cell lines. Fluorescence images of DOX drug after BEL-7402 and HepG2 cells incubated with Dex-PLGA/DOX/SPIO and A54-Dex-PLGA/DOX/SPIO micelles (drug content were 2.5 μg mL^−1^) for 1, 4, and 6 h, respectively. *In vitro* cytotoxicity of blank micelles against BEL-7402 **(B)**, HepG2 **(C)** and LO2 **(D)** cells. Data represent the mean ± standard deviation (n = 3).

Fluorescence images of DOX drug after BEL-7402 and HepG2 cells incubated with Dex-PLGA/DOX/SPIO and A54-Dex-PLGA/DOX/SPIO micelles for 1, 4, and 6 h were showed in Fig. 3A. The anti-tumor drug, DOX, could be internalized into tumor cells carried by the micelles and the uptake was time dependent. The DOX accumulation in BEL-7402 cells by A54-Dex-PLGA/DOX/SPIO micelles was larger than Dex-PLGA/DOX/SPIO micelles while there were no obvious difference among the micelles in HepG2 cells. And barely accumulation in LO2 cells was observed.

### *In vitro* anti-tumor activity of DOX and SPIO-loaded micelles

**Figure 4:**
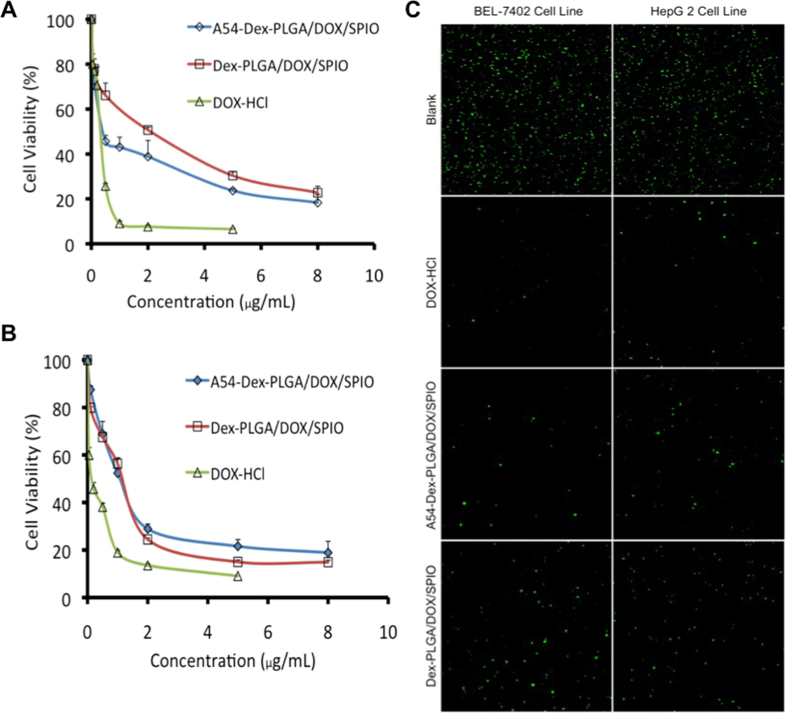
*In vitro* cytotoxicity of DOX/SPIO-loaded micelles and DOX • HCl against BEL-7402 **(A)** and HepG2 cells **(B)**. Data represent the mean ± standard deviation (n = 3). **(C)** BEL-7402 and HepG2 cells treated with DOX • HCl, Dex-PLGA/DOX/SPIO and A54-Dex-PLGA/DOX/SPIO micelles. Cells were stained with calcein acetoxymethyl ester (green) for visualization of live cells.

*In vitro* anti-tumor activity of DOX/SPIO-loaded micelles were evaluated using MTT method and colorimetric cell viability assay. Firstly, the cytotoxicities of SPIO-loaded micelles were determined using BEL-7402 (Fig. 3B), HepG2 (Fig. 3C), and LO2 (Fig. 3D) cell lines as model tumor cells. Cell viability was still higher than 80% when the concentration of Fe_3_O_4_ was 10 μg mL^−1^ (polymer was 1 mg mL^−1^), which indicated the SPIO-loaded micelles had relaively low cytotoxicity and high biocompatibility for both tumor cells and normal cells. *In vitro* cytotoxicity of DOX and DOX/SPIO-loaded micelles and DOX • HCl against BEL-7402 and HepG2 cells were shown in Fig. 4A,B and the 50% cellular growth inhibitions (IC_50_) were shown in Table 3. After DOX loading, the cytotoxicities of micelles increased distinctly, especially the A54-Dex-PLGA/DOX/SPIO micelles against BEL-7402 cells. The IC_50_ value was 0.56 μg mL^−1^, which increased more than 2-folded compared with that of Dex-PLGA/DOX/SPIO micelles (1.24 μg mL^−1^). However, the Dex-PLGA/DOX/SPIO micelles manifested similar cytotoxicities against HepG2 cells, and the IC_50_ was between 0.8~1.1 μg mL^−1^. This can be explained by the specific uptake of A54-Dex-PLGA/DOX/SPIO micelles to BEL-7402 cells and the non- specific interaction between Dex-PLGA/DOX/SPIO micelles and the tumor cells.

Colorimetric cell viability assay was used to investigate the cytotoxicities of DOX and DOX/SPIO-loaded micelles more directly. Figure 4C illustrated the results of DOX • HCl and DOX/SPIO-loaded micelles utilizing calcein AM stain with BEL-7402 and HepG2 cells for visualization of live cells. For the BEL-7402 cells, significantly less cells were lived (stained green by calcein AM) due to the treatment of A54-Dex-PLGA/DOX/SPIO micelles compared with Dex-PLGA/DOX/SPIO micelles, while no difference of HepG2 cells was observed between A54-Dex-PLGA/DOX/SPIO and Dex-PLGA/DOX/SPIO micelles treatment. These *in vitro* anti-tumor activity results suggested that A54-Dex-PLGA/DOX/SPIO micelles could efficiently carry drugs into tumor cells and significantly increase the cytotoxicities of micelles. Dex-PLGA coupled with homing peptides A54 was endowed the specific affinity to corresponding BEL-7402 cells *in vitro*.

### *In vitro* MR imaging of DOX and SPIO-loaded micelles

**Figure 5:**
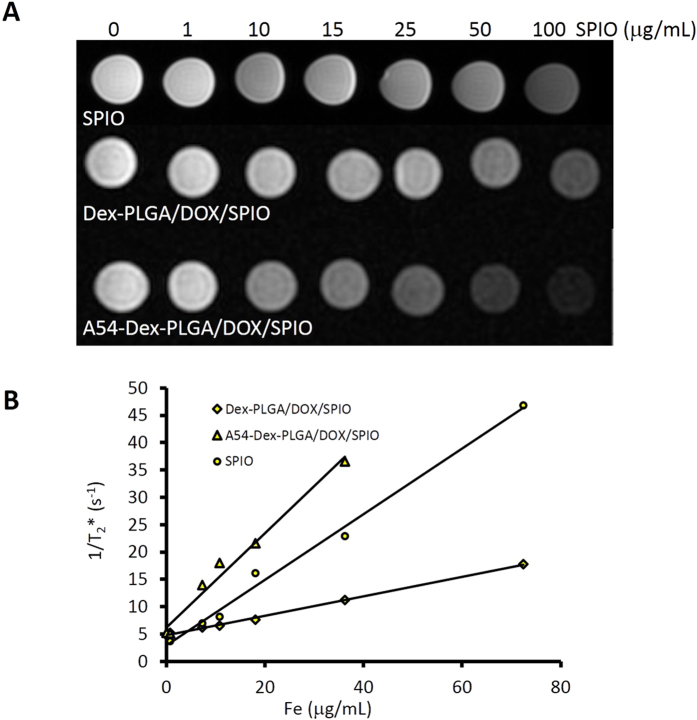
T_2_*-weighted images of bare SPIO, Dex-PLGA/DOX/SPIO micelles and A54-Dex-PLGA/DOX/SPIO micelles at different iron concentration **(A)**, and the chart of 1/T_2_* values changing with Fe concentration **(B)**.

Magnetic properties were important paramaters for an MRI contrast agent. As shown in Fig. 5A, the T_2_-weighted MRI of bare SPIO, A54-Dex-PLGA/DOX/SPIO and Dex-PLGA/DOX/SPIO micelles at a 3.0 T clinical MRI instrument presented obvious color change with a variation of Fe_3_O_4_ concentration. It was found that bare SPIO and DOX/SPIO-loaded micelles exhibited negative contrast enhancement as the concentration increased from 0 to 100 μg mL^−1^. The relaxation rate, R_2_ = 1/T_2_, linearly proportional to the Fe concentration was shown in Fig. 5B. A54-Dex-PLGA/DOX/SPIO and Dex-PLGA/DOX/SPIO micelles, as well as bare SPIO, both showed favorable contrast effect. The high relaxivity coefficient was prerequisite to be utilized as novel T_2_ negative contrast agent for sensitive MR imaging.

**Figure 6:**
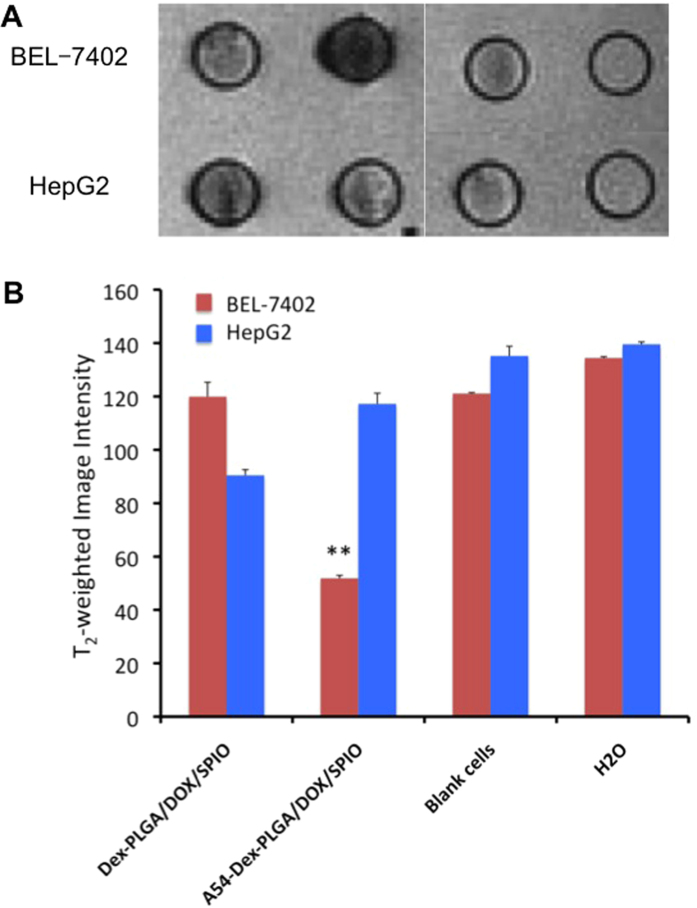
T_2_-weighted images of Dex-PLGA/DOX/SPIO micelles and A54-Dex-PLGA/DOX/SPIO micelles incubated with BEL-7402 and HepG 2 cells for 1 h **(A)** and the T_2_-weighted image intensity (**p < 0.01) **(B)**.

T_2_-weighted images of Dex-PLGA/DOX/SPIO and A54-Dex-PLGA/DOX/SPIO micelles incubated with BEL-7402 and HepG2 cells for 1 h and their intensity were shown in Fig. 6A,B. The T_2_-weighted image intensity of blank cells were similar with that of water, while after the uptake of micelles, the T_2_-weighted image intensity significant declined. The T_2_-weighted image intensity of BEL-7402 cells incubated with A54-Dex-PLGA/DOX/SPIO micelles was decreased most prominent, which existed significant differences compared with others (P < 0.01). This may due to the specific binding activity of A54-Dex-PLGA/DOX/SPIO micelles to BEL-7402 cells leading to the over expression of receptor on the cell surface and the receptor-mediated endocytosis to enhance the tumor cells uptake^19^.

### *In vivo* MR imaging of DOX and SPIO-loaded micelles

**Figure 7:**
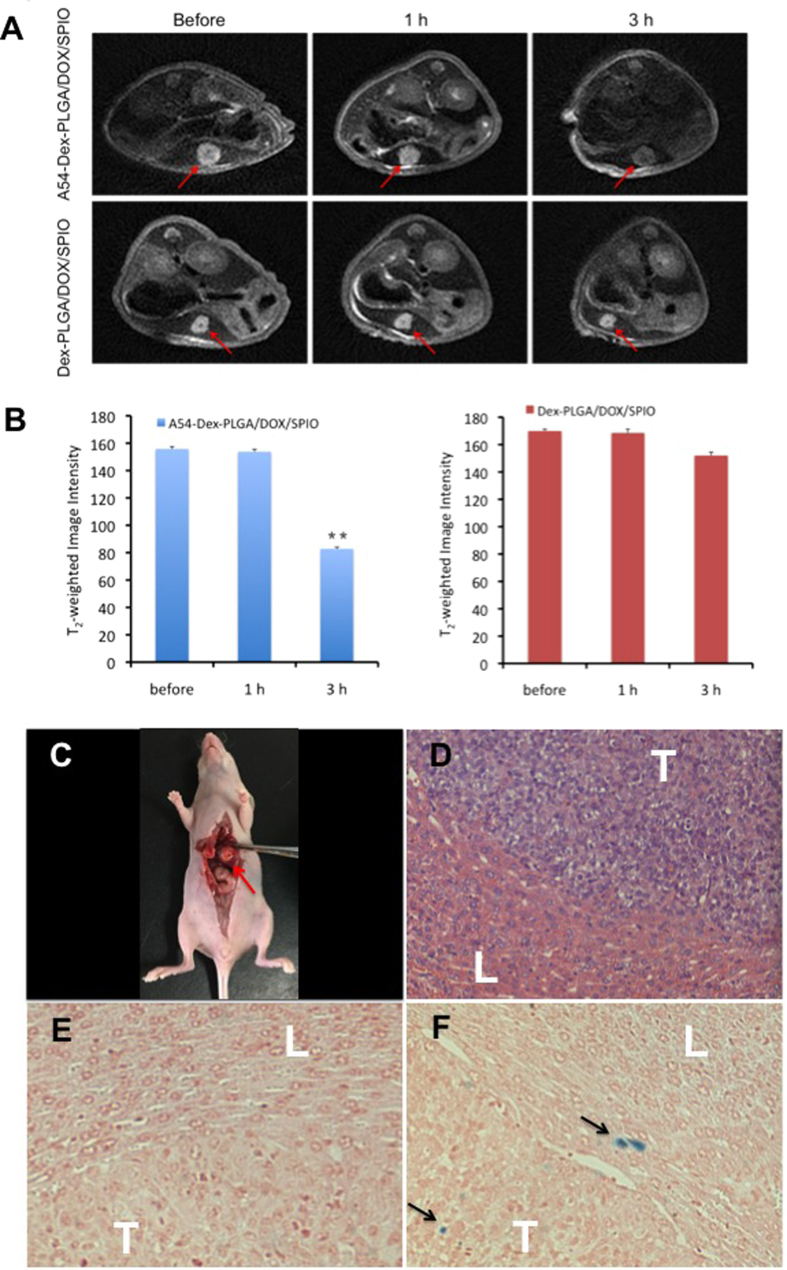
(**A**) T_2_WI images of nude mice bearing BEL-7402 orthotopic implantation tumor before contrast and after injection of Dex-PLGA/DOX/SPIO and A54-Dex-PLGA/DOX/SPIO micelles for 1 h and 3 h, respectively. The red arrow stands for tumors. **(B)** The T_2_-weighted image intensity (**p < 0.01). **(C)** The tumor model made in orthotopic implantation of BEL-7402 tumor. The red arrow stands for the tumor in liver; **(D)** Microscope image of HE stained live tissue section from nude mice bearing BEL-7402 tumor; Prussian blue stained liver-tumor tissue section of nude mice treated with Dex-PLGA/DOX/SPIO **(E)** and A54-Dex-PLGA/DOX/SPIO **(F)**, respectively. The black arrow stands for SPIO, “L” stands for the normal liver, “T” stands for the tumor.

Both *in vitro* cellular uptake and MR imaging studies indicated that A54-Dex-PLGA/DOX/SPIO micelles could target BEL-7402 cells. *In vivo* MR imaging of DOX/SPIO-loaded micelles were then examined using orthotopic implantation hepatoma models. Figure 7A,B showed the T_2_WI images of nude mice bearing BEL-7402 orthotopic implantation tumor before contrast and after injection of Dex-PLGA/DOX/SPIO and A54-Dex-PLGA/DOX/SPIO micelles and their T_2_-weighted image intensity. The results showed that the tumor image of post-injection of A54-Dex-PLGA/DOX/SPIO micelles for 3 h become darkened significantly compared with that of pre-injection of micelles and the T_2_-weighted image intensity of tumor was significantly decreased (P < 0.01), while no obvious difference was observed after injection of Dex-PLGA/DOX/SPIO micelles. These results concluded that the A54-Dex-PLGA/DOX/SPIO micelles enhanced the contrast in tumor tissue and might be used as a T_2_ negative contrast agent for MR imaging application.

To further verify the accumulation of iron at tumor tissue, the tissue slices were stained with haematoxylin-eosin (H&E) and Prussian blue. As shown in Fig. 7D, the tumor cells were larger, pleomorphic and had biger nucleus, abundant cytoplasm, which confirmed that the orthotopic implantation hepatoma models were successfully established. The accumulation of iron could be clearly observed in the tumor tissues of A54-Dex-PLGA/DOX/SPIO micelles injected group (Fig. 7E). In contrast, no apparent accumulation was detected in the Dex-PLGA/DOX/SPIO micelles injected group (Fig. 7F).

### *In vivo* anti-tumor activity of DOX and SPIO-loaded micelles

**Figure 8:**
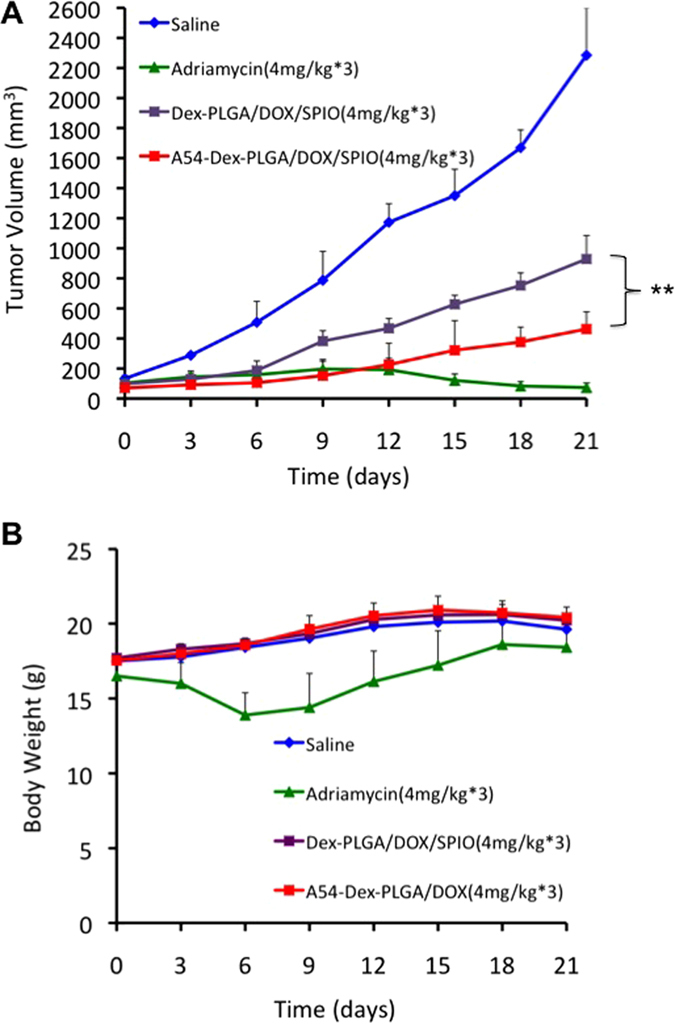
*In vivo* anti-tumor activities of Adriamycin and Dex-PLGA/DOX/SPIO, A54-Dex-PLGA/DOX/SPIO micelles in tumor bearing nude mice after i. v. injection. Mice tumor volume (**p < 0.01) **(A)** and mice body weight **(B)** changed within 21 days. Data represent the mean ± standard deviation (n = 4).

Adriamycin (commercial doxorubicin hydrochloride injection), DOX/SPIO-loaded micelles solution were injected through the tail vein into nude mice bearing BEL-7402 human hepatoma. The changes in the tumor volume were plotted as shown in Fig. 8A. Both Adriamycin and DOX/SPIO-loaded micelles treatments effectively suppressed tumor growth, while the tumor volume of saline group showed a sharply growth rate. After i.v. injection for 3 days, tumor volumes of nude mice treated with adriamycin and the DOX/SPIO-loaded micelles were significantly smaller than those treated with saline (p < 0.01), and after 9 days, p < 0.005. At the 12^th^ day, the tumor volumes of nude mice treated with adriamycin were smaller than those treated with A54-Dex-PLGA/DOX/SPIO micelles. This might be related to the slowly release after the drug reached the tumor tissues, which was caused by improving the targeting efficiency and avoiding drug leakage from drug-loaded micelles in circulation process. What is more, the tumor volumes of nude mice treated with A54-Dex-PLGA/DOX/SPIO micelles were consecutively smaller than those treated with Dex-PLGA/DOX/SPIO micelles. Tumor reduction between Dex-PLGA/DOX/SPIO and A54-Dex-PLGA/DOX/SPIO nanoparticles was significant. After 21 days, the tumors were harvested. The tumor inhibition rate of micelles treatments was 72.53% treated with A54-Dex-PLGA/DOX/SPIO micelles and 62.08% with Dex-PLGA/DOX/SPIO micelles. All the tumor inhibition values were larger than 60%, which could be considered effective treatment.

Figure 8B showed that the body weight of DOX/SPIO-loaded micelles treated and un-treated mice was continuously increased, which indicated the low toxicity and high biocompatibility of DOX/SPIO-loaded micelles. However, at the 3^rd^ day, the body weight of adriamycin group sharply declined, which revealed the high toxicity of this commercial doxorubicin hydrochloride injection.

These results indicated that A54-Dex-PLGA/DOX/SPIO micelles could target to the tumor tissue more efficiently, reduced toxicity and had better anti-tumor effect compared with Dex-PLGA/DOX/SPIO micelles. This is mainly because A54-Dex-PLGA/DOX/SPIO micelles successfully inherited the ability of Dex-PLGA graft to enter cells and the ability of biological molecules A54 peptides to specifically target liver cancer cells BEL-7402, and transported drugs into the tumor cells through the EPR effect and targeting molecules mediated endocytosis to obtain a better therapeutic effect.

#### Summary

A54-Dex-PLGA graft was designed and synthesized successfully via esterification reaction and could form polymeric micelles in aqueous solution for the delivery of doxorubicin and superparamagnetic iron oxide. A54 peptide modified and SPIO encapsulated had no significant effect on encapsulation efficiency, drug loading and *in vitro* release of doxorubicin. The A54-Dex-PLGA/DOX/SPIO micelles were effectively transported to the tumor tissue through the EPR effect, the targeting peptide A54 enhanced endocytosis of the micelles, then could achieve diagnostic, monitoring and therapeutic purposes. The assay of *in vitro* and *in vivo* anti-tumor activities indicated that A54-Dex-PLGA/DOX/SPIO micelles could reduce the toxicity and revealed better therapeutic effects compared with Dex-PLGA/DOX/SPIO micelles. The research suggests that, with the mediation of homing peptide A54, the A54-Dex-PLGA carrier has promising potential in special tumor targeting, efficient therapy for tumor, systematic toxicity reduction and tumor targeting molecular imaging for MR. A multifunctional graft micelle delivery system A54-Dex-PLGA/DOX/SPIO was successfully constructed for tumor diagnosis, detection and therapy.

## Materials and Methods

### Synthesis of A54 Peptide Functionalized Dextran-g-PLGA (A54-Dex-PLGA)

With the dehydrating agent DCC and the catalyst DMAP, Dex-PLGA graft was synthesized by esterification reaction between the carboxyl group of PLGA and the hydroxyl group of dextran. Briefly, PLGA, DCC and DMAP (PLGA:DCC:DMAP = 1:3:0.3, mol:mol) were dissolved in anhydrous DMSO and stirred at 60 °C for 30 min to activate the carboxyl group of PLGA. Dex (Dex:PLGA = 1:15, mol:mol) was added to react for 48 h then centrifugation. After dialysis against pure water for 48 h, the supernatant was lyophilized. The product was further dispersed in acetone (20 mg mL^−1^) and then lyophilized again.

A54-Dex-PLGA graft was synthesized by esterification reaction between the carboxyl group of A54 peptide and the dissociative hydroxyl group of dextran with the utilization of (Boc)_2_O to protect the amino of A54 peptide. (Boc)_2_O and A54 peptide were dissolved in anhydrous DMSO ((Boc)_2_O:A54 = 1:1, mol:mol) and stirred with light protection at room temperature for 12 h. Then Dex-PLGA, DCC and DMAP in DMSO were added into the reaction system with stirring for 48 h at room temperature. After 2 M HCl was added to remove Boc protecting group, the product was dialyzed against pure water for 48 h and then lyophilized.

### Preparation of SPIO-loaded Dex-PLGA and A54-Dex-PLGA micelles, DOX/SPIO-loaded Dex-PLGA and A54-Dex-PLGA micelles

SPIO-loaded Dex-PLGA and A54-Dex-PLGA micelles were prepared using emulsion-solvent evaporation method. Briefly, polymers (10 mg) were dissolved in 5 mL deionized water and 100 μL SPIO (5 nm, 5 mg/mL in toluene, Sigma-Aldrich Chemical (USA)) was added under ultrasonic for 10 min (SPIO:polymers = 5%, w/w). Rotary evaporation procedure was applied to remove toluene and the temperature was controlled at 40 °C. Then the SPIO loaded micelles (Dex-PLGA/SPIO and A54-Dex-PLGA/SPIO) were obtained.

For the preparation of DOX/SPIO-loaded Dex-PLGA and A54-Dex-PLGA micelles, Doxorubicin base was firstly obtained by reaction of doxorubicin hydrochloride (DOX-HCl) with double mole triethylamine (TEA) in DMSO overnight. Polymers (10 mg) and DOX were dissolved in 1 mL DMSO solution (DOX:polymers = 5%, w/w) under magnetic stirring in room temperature. After the mixture solution was dialyzed against pure water (MWCO = 3.5 kDa) for 24 h, the products were centrifuged at 4000 rpm for 10 minutes to obtain the DOX-loaded micelles solution. Then SPIO was added as previously described to obtain DOX/SPIO-loaded Dex-PLGA and A54-Dex-PLGA micelles (Dex-PLGA/DOX/SPIO and A54-Dex-PLGA/DOX/SPIO).

### Physicochemical characteristics of polymers and micelles

NMR spectrometer (AC-80, Bruker Biospin, Germany) was used to obtain the ^1^H NMR spectra of chemicals. Dimethyl sulfoxide-d_6_ was the solvent for NMR measurement.

The critical micelle concentration (CMC) of Dex-PLGA and A54-Dex-PLGA was evaluated by fluorescence measurement using pyrene as a probe. The excitation wavelength was set at 337 nm, the excitation slit at 10 nm and emission slit at 2.5 nm. The intensities of the emission at a wavelength range of 300–470 nm were recorded on a fluorescence spectrophotometry (F-2500, Hitachi Co., Japan). The concentration of polymer solution was ranging from 10^−3^ to 1.0 mg mL^−1^. Then the intensity ratio of the first peak (I_1_, 374 nm) to the third peak (I_3_, 385 nm) was analyzed for determination of CMC.

The size and distribution of polymer micelles were determined by dynamic light scattering using a ZETASIZER (3000HS, Malvern Co.,UK). The morphological examinations were performed by a transmission electron microscopy (TEM, JEOL JEM-1230, Japan). The samples were dropped on copper grids and stained with 2% (w/v) phosphotungstic acid for viewing.

The solution of SPIO-loaded micelles were lyophilized and magnetic properties of the powders were measured on a vibrating sample magnetometer (a Physical Property Measurement System from Quantum Design, MPMS-XL-5, CA, USA) with a maximum applied field of 1.8 T at room temperature.

### Determination of drug encapsulation efficiency and drug loading

The DOX content in micelles was determined using a fluorescence spectrophotometry (F-2500, Hitachi Co., Japan). DOX-loaded micelles solution was diluted 10-fold by DMSO and the fluorescence intensity was measured. The excitation wavelength was set at 505 nm while the emission wavelength was at 565 nm. The excitation and the emission slit was 5.0 nm. The drug encapsulation efficiency (EE%) and drug loading (DL%) were calculated using formula 2 and 3 below, respectively:


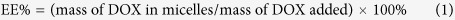



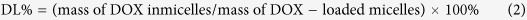


### *In vitro* DOX release from DOX/SPIO-loaded micelles

*In vitro* drug release tests were conducted using the dialysis method. 3 mL DOX-loaded, DOX/SPIO-loaded micelles solution was put into a dialysis membrane (MWCO:3.5 kDa) and added into a plastic tube containing 20 mL phosphate-buffered saline (PBS) solution at pH 7.2. Besides, to reflect intracellular acidic environment of the tumor, the drug release behavior of free DOX and A54-Dex-PLGA/DOX at pH 5.5 PBS were performed simultaneously. The tests were carried out in an incubator shaker (37 °C, 70 rpm). At predetermined time intervals (0.5, 1, 2, 4, 6, 8, 12, 24, 36, 48, 72 h), all of the medium out of the dialysis membrane were withdrawn and replaced with the fresh PBS. DOX content was determined by a fluorescence spectrophotometer (F-2500, Hitachi Co., Japan). All drug release tests were performed three times.

### Cell culture

BEL-7402, HepG2 and LO2 cell lines were maintained in DMEM supplemented with 10% (v/v) FBS (fetal bovine serum) and penicillin/streptomycin (100 U mL^−1^, 100 U mL^−1^) at 37 °C in a humidified atmosphere containing 5% CO_2_. Cells were subcultured regularly using trypsin/EDTA.

### Cellular competitive uptake

HepG2 and LO2 cells were stained with Fluorescent Cell Linker PKH67 (Sigma-Aldrich; St. Louis, MO USA) followed to the protocol, which could incorporate into cell membrane with no influence with biological activity^40^,^41^. Briefly, cells were re-suspended in 500 μL Diluent C and then 500 μL PKH67 dye (1 μL PKH67 in 1 mL Diluent (C) was added. After incubation with PKH67 at room temperature for 10 min, the cells were centrifuged at 4000 rmp for 10 min. The cells were washed 2 more times with medium to remove the unbound dye and re-suspended to the desired concentration.

The PKH67 labeled HepG2 or LO2 cells were co-cultured with BEL-7402 cells respectively in a same well of 24-well plate and incubated for 24 h. Then the Rhodamine B Isothiocyanate labeled micelles (RITC:polymer = 2:1, mol:mol) were added and incubated with cells for 1 h. After the cells were washed three times, the cellular uptake was observed by a confocal microscopy (BX61W1-FV1000, Olympus, Japan).

### Specific internalization of DOX/SPIO-loaded micelles

BEL-7402, HepG2 and LO2 cell lines were used to study the internalization of Dex-PLGA/DOX/SPIO and A54-Dex-PLGA/DOX/SPIO micelles. Cells were seeded at 3.0 × 10^4^ cells per well in a 24-well plate (Nalge Nunc International, Naperville, IL, USA) and incubated for 24 h to attach. And then 100 μl of DOX and SPIO-loaded micelles (DOX content was 2.5 μg mL^−1^) was added, followed by further incubation for 1, 4 and 6 h. After the cells were washed with PBS three times, the cellular uptake was examined under fluorescence microscopy (Leica DM4000 B, Leica, Solms, Germany).

### *In vitro* anti-tumor activity

The cytotoxicity of DOX • HCl, SPIO-loaded micelles and DOX/SPIO-loaded micelles against BEL-7402, HepG2 and LO2 cells was evaluated by MTT assay. Briefly, 1.0 × 10^4^ cells per well were placed in a 96-well microtiter plate (Nalge Nunc International, Naperville, IL, USA) and incubated for 24 h. Then the cells were incubated with a series of concentration of micelles for another 48 h. After that, cells were incubated with 20 μL MTT solution (5 mg mL^−1^) for further 4 h at 37 °C. Finally, the product was dissolved by 100 μL DMSO and the absorbance was determined at 570 nm using an automatic reader (Bio-Rad, Model 680, USA). All the experiments were repeated three times. The 50% cellular growth inhibitions (IC_50_) with 48 h were determined.

Colorimetric cell viability assay^42^,^43^ was further used to investigate the cytotoxicity of DOX • HCl, DOX/SPIO-loaded micelles against BEL-7402 and HepG2 cells. The drug concentration of DOX • HCl, DOX/SPIO-loaded micelles were same as the IC_50_ value of A54-Dex-PLGA/DOX/SPIO micelles. After a 24 h drug exposure, cells were stained with 100 μL calcein AM (Invitrogen, Carlsbad, CA) (0.5 μL dissolved in 1 mL PBS) for 30 min at room temperature. Cells were observed using a fluorescence microscope (Leica DM4000 B, Leica, Solms, Germany).

### *In vitro* 3.0 T MR imaging of DOX/SPIO-loaded micelles

Magnetic resonance imaging (MRI) was used to assess the magnetic properties of bare SPIO, Dex-PLGA/DOX/SPIO and A54-Dex-PLGA/DOX/SPIO micelles. Bare SPIO and DOX/SPIO-loaded micelles solutions were diluted at various Fe_3_O_4_ concentrations of 100, 50, 25, 15, 10, 1, 0 μg mL^−1^. R_2_ relaxivities, defined as 1/T_2_ with units of s^−1^, of micelles solution were measured at room temperature with a 3.0 T clinical MRI system (GE, Discovery MR 750, USA). The parameters were as followed:FOV = 180 × 180 mm, TR = 2000 ms, TE = 12 ms, slice thickness = 3.0 mm, number of slices = 8.

BEL-7402 and HepG2 cells were incubated with Dex-PLGA/DOX/SPIO and A54-Dex-PLGA/DOX/SPIO micelles for 1 h. After the medium was discarded and the cells were washed with PBS three times, the cells were mixed with 200 μL agarose gel and stored at room temperature. The T_2_-weighted images were acquired using a 3.0 T clinical MRI system (GE, Discovery MR 750, USA). The parameters were as the above description.

### *In vivo* MR imaging studies

All animal procedures were approved by the Zhejiang University Institutional Animal Care and Use Committee and the methods were performed in accordance with the National Institutes of Health (NIH, USA) guidelines. A BALB/C + nu/F1 nude mice about 6–8 weeks old was transplanted with BEL-7402 cells to induce orthotopic implantation hepatoma models. Briefly, a middle-line incision was made in the abdomen, and the liver was partially pulled out to expose the liver. 1 mm^3^ tumor tissue was implanted into the liver parenchyma. The exteriorized organs were then returned to the peritoneal cavity, and the incision in the body wall and skin sutured.

Two weeks after surgery, animals were studied for T_2_-weighted MR imaging. 0.2 mL Dex-PLGA/DOX/SPIO or A54-Dex-PLGA/DOX/SPIO micelles solution (SPIO content was 100 μg mL^−1^) was injected via tail vein. The mice were anesthetized and imaged at the predetermined time (0, 1, 3 h) after the injection using a 3.0 T clinical MRI system (GE, Discovery MR 750, USA). The parameters were as follows:FOV = 60 × 60 mm, TR = 3000 ms, TE = 78.6 ms, slice thickness = 2.0 mm, number of slices = 10.

### Histological analysis

Animals were sacrificed to remove the liver for histological analysis (Fig. 7C). The tissues were fixed with 10% neutral buffered formalin and embedded in paraffin then sectioned. The sections were stained with haematoxylin-eosin (H&E) for histological analysis. Prussian blue staining was carried out to visualize accumulation of iron in the tissues. The microscopic images were photographed by a fluorescence microscopy (Leica, Germany).

### *In vivo* anti-tumor activity

To investigate the *in vivo* anti-tumor activity of DOX/SPIO-loaded micelles, BALB/C + nu/F1 nude mice about 6–8 weeks old were transplanted with BEL-7402 cells and drug injection via tail vein was started when the tumor volume reached approximately 100 mm^3^. Mice were divided into four groups randomly, four mice in each group. The negative control group was injected with 0.2 mL 0.9% saline solution and the positive group with 0.2 mL adriamycin (commercial doxorubicin hydrochloride injection, 4.0 mg kg^−1^ body weight). The third and fourth groups were injected with 0.2 mL Dex-PLGA/DOX/SPIO and A54-Dex-PLGA/DOX/SPIO micelles (4.0 mg kg^−1^ body weight). All groups were dosed once every other day, for 3 times (red arrow in Fig. 8A).

The size of the tumor and the body weight of each mouse were monitored every 3 days thereafter. After 21 days, the nude mice were sacrificed, and the tumor weight was measured.

Tumor volume was calculated using the formula, a^2^ × b/2, where a was the smallest and b was the largest diameter. The inhibition of tumor growth (%) was calculated using formula 4 below:


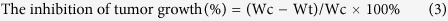


Where, Wc is the average tumor weight of controlled group, Wt is the average tumor weight of treated group. The treatment is considered effective if the value of the inhibition of tumor growth is higher than 60%.

## Additional Information

**How to cite this article**: Situ, J.-Q. *et al.* Multifunctional SPIO/DOX-loaded A54 Homing Peptide Functionalized Dextran-g-PLGA Micelles for Tumor Therapy and MR Imaging. *Sci. Rep.* **6**, 35910; doi: 10.1038/srep35910 (2016).

## Figures and Tables

**Table 1:** Particle diameter and saturation magnetization values of Dex-PLGA, A54-Dex-PLGA micelles and SPIO-loaded micelles.

Material	Size (nm)	PI	Ms (emu/g)
A54-Dex-PLGA	153	0.781	—
Dex-PLGA	138	0.610	—
A54-Dex-PLGA/SPIO	83	0.380	21
Dex-PLGA/SPIO	40	0.329	31

**Table 2:** Particle diameter, entrapment efficiency and drug loading of Dex-PLGA/DOX, A54-Dex-PLGA/DOX micelles and Dex-PLGA/DOX/SPIO, A54-Dex-PLGA/DOX/SPIO micelles.

Material	Drug feeding (%, w/w)	Size (nm)	PI	EE (%)	DL (%)
A54-Dex-PLGA/DOX	5	116	0.121	75.31	3.59
Dex-PLGA/DOX	5	107	0.129	75.37	3.59
A54-Dex-PLGA/DOX/SPIO	5	97	0.146	79.98	3.81
Dex-PLGA/DOX/SPIO	5	87	0.164	79.19	3.77

**Table 3:** IC_50_ value of DOX/SPIO-loaded micelles and DOX • HCl against BEL-7402 and HepG2 cells (μg mL^−1^).

Cell Line	DOX • HCl	A54-Dex-PLGA/DOX/SPIO	Dex-PLGA/DOX/SPIO
BEL-7402	0.2379	0.5589	1.2402
HepG2	0.1276	1.0685	0.8382
